# Varicella zoster virus transmission dynamics in Vojvodina, Serbia

**DOI:** 10.1371/journal.pone.0193838

**Published:** 2018-03-05

**Authors:** Snežana Medić, Michalis Katsilieris, Zagorka Lozanov-Crvenković, Constantinos I. Siettos, Vladimir Petrović, Vesna Milošević, Snežana Brkić, Nick Andrews, Milan Ubavić, Cleo Anastassopoulou

**Affiliations:** 1 Center for Disease Control and Prevention, Institute of Public Health of Vojvodina, Novi Sad, Serbia; 2 Faculty of Medicine, University of Novi Sad, Novi Sad, Serbia; 3 School of Applied Mathematics and Physical Sciences, National Tehnical University of Athens, Athens, Greece; 4 Department of Mathematics and Computer Science, Faculty of Science, University of Novi Sad, Novi Sad, Serbia; 5 Clinic for Infectious Diseases, Clinical Center of Vojvodina, Novi Sad, Serbia; 6 Statistics, Modelling, and Economics Department, National Infections Services, Public Health England, London, United Kingdom; 7 Medlab, Health Institution, Novi Sad, Serbia; 8 Division of Genetics, Cell and Developmental Biology, Department of Biology, University of Patras, Patras, Greece; Laboratoire National de Santé, LUXEMBOURG

## Abstract

This study aimed at establishing baseline key epidemiological parameters for varicella zoster virus (VZV) infection in Vojvodina, Serbia, with the ultimate goal to quantify the VZV transmission potential in the population. Seroprevalence data generated during the first large cross-sectional VZV serosurvey were modelled, using a two-tiered modelling approach to calculate age-specific forces of infection (FOI), the basic reproduction number (*R*_0_) and herd immunity threshold (*H*). Seroprevalence and modelling data were compared with corresponding pre-vaccination epidemiological parameters from 11 countries participating in the European Sero-Epidemiology Network 2 (ESEN2) project. Serbia fits into the general dynamic VZV transmission patterns in Europe in the pre-vaccine era, with estimated *R*_0_ = 4.12, (95% CI: 2.69–7.07) and *H* = 0.76 (95% CI: 0.63–0.86). The highest VZV transmission occurs among preschool children, as evidenced by the estimation of the highest FOI (0.22, 95% CI: 0.11–0.34) in the 0.5–4 age group, with a peak FOI of 0.25 at 2.23 years. Seroprevalence was consistently lower in 5–14 year-olds, resulting in considerable shares of VZV-susceptible adolescents (7.3%), and young adults (6%), resembling the situation in a minority of European countries. The obtained key epidemiological parameters showed most intense VZV transmission in preschool children aged <4 years, justifying the consideration of universal childhood immunization in the future. National immunization strategy should consider programs for VZV serologic screening and immunization of susceptible groups, including adolescents and women of reproductive age. This work is an important milestone towards the evaluation of varicella immunization policy options in Serbia.

## Introduction

Varicella (chickenpox), the primary disease caused by varicella zoster virus (VZV) is normally a mild childhood illness with characteristic vesicular rash [1]. Complications of the infection are associated with age extremes, pregnancy and immunocompromised conditions [2]. In Europe, the estimated burden of varicella in the pre-vaccine era was significant as reflected by the >5 million new cases annually, of which more than half sought physician consultation and ~20,000 led to hospitalizations and up to 80 deaths [3]. Although infected adults are at higher risk of hospitalization and death, varicella mainly affects previously healthy children, underscoring the importance of not dismissing varicella as a disease of little clinical relevance [2,3]. After chickenpox, VZV persists asymptomatically in the body to reactivate later causing a secondary disease, herpes zoster (HZ, shingles), typically in older individuals with impaired cellular immunity [1,3].

Live attenuated varicella vaccines proved to be safe and efficacious in preventing varicella [4]. However, uncertainties over the potential impact of varicella immunization on the epidemiology of VZV infections pose obstacles to reaching a consensus on vaccination policy [5]. Most European Union/European Economic Area (EU/EEA) countries have varying recommendations for varicella vaccination. Only a few countries have opted for routine childhood immunization, while most countries (17/29) recommend vaccination solely for susceptible teenagers and/or susceptible risk groups [4]. In spite of the high incidence of varicella and the availability of vaccine, immunization against varicella has been just recently introduced into national legislation, in the Republic of Serbia. Immunization against HZ is recommended by the same law [6,7].

Population immunity against vaccine-preventable infections may be estimated through cross-sectional studies of antibody prevalence [8]. To allow for international comparisons, the European Sero-Epidemiology Network (ESEN2) introduced standardized serological surveillance to a number of vaccine-preventable infections, including VZV [9,10]. The VZV ESEN2 data from 11 participating countries, together with newly available serology for Poland and Italy, were re-analyzed by Santermans *et al*. who corroborated that primary VZV infection most often occurs in early childhood across Europe, but with a substantial variation in the country-specific transmission potential [10,11].

Serological data may be modelled to obtain unbiased estimates of key parameters in infectious disease epidemiology [12]. Three pivotal parameters need to be estimated to quantify the VZV transmission potential in a population: the force of infection (FOI), basic reproduction number (*R*_*0*_) and herd immunity threshold (*H*) [10,11]. The FOI, symbolized by [*λ*], is the rate at which susceptible individuals acquire infection. This parameter, which reflects the contagiousness of an infectious agent, may be estimated from seroprevalence data and used to compare the transmission rate of the infection between different age groups. *R*_*0*_ represents the number of secondary cases that result from the introduction of a single infectious case in a totally susceptible population during the infectiousness period. To eliminate endemic transmission of infection, and thus eradicate the disease, a proportion of the population, the herd (*H*), needs to be immunized [10].

The elucidation of the interplay between virus transmission patterns and the immune status of the population using traditional serological studies in conjunction with state-of-the-art modelling approaches can guide vaccination policy most efficiently. Herein, we use the age-specific VZV seroprevalence data we have recently obtained (data not shown) to estimate key epidemiological parameters. Seroprevalence and modelling data were assessed comparatively to those obtained from 11 countries participating in ESEN2 [10], with the ultimate goal to design the most effective immunization strategy in Serbia. This work constitutes an important milestone towards this goal.

## Materials and methods

### Seroprevalence data

In this article, we analyze the VZV seroprevalence data from Vojvodina, Serbia that had been obtained during the 2015–2016 serosurvey (S1 File) using the ESEN2 data on VZV published by Nardone *et al*. [10] as a basis for comparisons. At the time of sera collection (between 1995 and 2003), universal VZV immunization had not been introduced in any of the eleven countries participating in ESEN2. Serbian seroprevalence data were generated from the testing of 3570 anonymised residual diagnostic sera from patients of all ages (age range: 29 days-83 years, with 52 samples from children <6 months), and predominantly (59.4%) from urban areas, collected as part of routine care in the Autonomous Province of Vojvodina (S1 File). Vojvodina is a northern Serbian province with a population of about two million (~27% of the population of Serbia excluding Kosovo), extending over an area of 21,506 km^2^. Immunocompromised individuals and recent recipients of blood and blood products were excluded. Available information for each patient included the following: sex, age, area of residence in Vojvodina and sample collection date. The age stratification of the sera was according to the specifications of ESEN2 [10]. Accordingly, ~100 samples were collected for each year band in the age group 0–19 years and 200 samples for each of the age groups ≥20 years (20–24, 25–29, 30–34, 35–39, 40–49, 50–59, and ≥60), with about equal numbers of samples by gender. Samples were tested using anti-VZV ELISA (IgG) [EUROIMMUN AG, Germany] according to the manufacturer’s guidelines. Obtained results were standardized into common, ESEN2 units, with equivocals (low positives) included as seropositives [9,10]. Written informed consent of study participants, or their parents or legal guardians if they were <15 years, was obtained. The study was approved by the Medical Ethics Committee of the Institute of Public Health of Vojvodina, in accordance to the Declaration of Helsinki of 1975, as revised in 2008.

### Estimation of age-specific force of VZV infection

To overcome the limitations of any given method for the estimation of the rate at which susceptible individuals acquire infection (i.e. the FOI or *λ*), we used two different, but complementary approaches: the so called catalytic model assuming a piecewise constant force of infection and Farrington's exponentially damped linear model. The latter approach allows the estimation of such additional key epidemiological parameters as *R*_*0*_ and *H*, on the basis of obtained age-specific FOI. Moreover, international comparisons within the European region, originally undertaken by Nardone *et al*. [10], were thus rendered possible. Passive immunity of infants, acquired by the transfer of maternal antibodies, declines rapidly by the age of 6 months, leading to low FOIs in the <1 year age group [4,13]. To avoid this confounding factor, seroprevalence data from infants <1 year (N = 100, anti-VZV seropositivity = 75%) were excluded from further analysis. Secondly, our data do not represent a random sample of the population, which should be considered when interpreting the results.

#### Farrington's exponentially damped linear catalytic model

A catalytic model, as applied by Farrington [14] and Socan *et al*. [13] on the basis of Griffiths’ study [15], was used for the estimation of age-specific FOIs. Age-specific FOIs were estimated by modelling the average proportion of seropositive subjects *F*(*x*) in the respective age group *x*, where the model for *F*(*x*) is based on the so-called catalytic model. *F*(*x*) of childhood diseases has been shown to be well fitted by the equation:

$$F(x)=1-\exp\left(\frac{a}{b}xe^{-bx}+\frac{1}{b}\left[\frac{a}{b}-c\right]\left[e^{-bx}-1\right]-cx\right)$$

where *a*, *b* and *c* are the parameters to be estimated (*a*, *b*, *c* ≥ 0). After a graphical validation of the appropriateness of the model for our data, the model was fitted using the nonlinear least squares method [13]. Modelling and data analyses were performed using Statistica 13 [16].

The FOI is defined as the negative derivative of the logarithm of the survival function *S(x) = 1-F(x)* [12,14], i.e. as

$$\lambda \left(x\right)=-\frac{d}{dx}\ln(1-F\left(x\right)),$$

thus yielding the following expression:

$$\lambda (x)=axe^{-bx}-ce^{-bx}+c.$$

#### Catalytic model assuming a piecewise constant force of infection

Age-specific FOIs were calculated for three different age groups (0.5–4, 5–9 and ≥10 years). For our calculations we assumed closed populations of *N* persons, mortality as type I developed countries with a life expectancy of *L* = 75 years and passive immunity in all infants until the age of *A* = 0.5 years [17]. Here, the FOIs were estimated by maximizing the log-likelihood function [10,18–20].

$$L=\sum_{j=1}^{n}M_{x_{j}}\log\left[F\left(x_{j}\right)\right]+\left(N_{x_{j}}-M_{x_{j}}\right)\log\left[1-F\left(x_{j}\right)\right]$$

For each age group *j*, *x*_*j*_ denotes the average age in group *j*, $N_{x_{j}}$ denotes the total number of persons and $M_{x_{j}}$ the number of seropositive persons in group *j*. The function *F(x)* represents the estimated proportion of individuals at age *x* (in years) who are anti-VZV-positive, and is given by the following expression [20]:

$$F(x)=\left\{ \begin{array}{l} \;1,\;x<0.5\\ \;1-\exp(-l_{1}x),\;0.5\leq x<5\\ \;1-\exp\left({-l}_{1}(5)-l_{2}(x-5)\right),\;5\leq x<10\\ \;1-\exp({-l}_{1}(5)-l_{2}(9-5)-l_{3}(x-9)),\;10\leq x \end{array}\right.$$

Where *l*_*1*_, *l*_*2*_, *l*_*3*_ are constants. Estimations of the constants were obtained by maximizing the log-likelihood *L*.

The FOIs were computed as above by the negative derivative of the logarithm of the corresponding survival functions. The upper and lower 95% confidence intervals (CI) for each age-specific FOI were obtained assuming a normal approximate distribution using the deviance between the saturated and the fitted model.

#### Estimation of basic reproductive number (*R*_*0*_)

To calculate *R*_*0*_ we proceeded as follows. It has been shown that the average age-specific force of infection *λ*(*x*) in the discrete age intervals [*a*_*i*_ *a*_*i*+1_) are given by [19]:

$$\lambda_{i}=\frac{ND}{L}\sum_{j=1}^{J}\beta_{ij}[\exp\left(-\sum_{k=1}^{j-1}\lambda_{k}(a_{\left[k+1\right]}-a_{\left[k\right]})\right)-\exp\left(-\sum_{k=1}^{j}\lambda_{k}(a_{\left[k+1\right]}-a_{\left[k\right)})\right)]$$

where *β*_*ij*_ denotes the effective contacts per person of an individual of age class *j* with a person of age class *i*, per year, and *D* is the mean duration of infectiousness; *a*_*[*__*1*__*]*_ *= A* and *a*_*[J+1]*_ *= L* [15]. Note that the estimation of the FOI is based on the maximization of the maximum likelihood function and hence an explicit knowledge of *N* is not required; instead, the ratio $\frac{ND}{L}$ is used.

The transmission rates *β*_*ij*_ make up a *J × J* matrix, the so-called “Who Acquires Infection From Whom”, WAIFW-matrix. Estimating the WAIFW matrix, reflecting the heterogeneous mixing between the three different age groups, we employed the age-specific transmission rates *β*_*ij*_ as described below [10,21].

                            *<* 5 years     5 − 9 years       ≥ 10 years

*<* 5 years             *β*_1_                 *β*_3_                     *β*_3_

5 − 9 years         *β*_3_                 *β*_2_                     *β*_3_

≥ 10 years          *β*_3_                 *β*_3_                     *β*_3_

*R*_*0*_ is defined by the dominant eigenvalue of the *J × J* next generation matrix with elements *(i*, *j = 1*… *J)* [19,22]:

$$\frac{ND}{L}(a_{\left[i+1\right]}-a_{\left[i\right]})\beta_{ij}$$

95% CI were also estimated based on the corresponding FOI.

#### Estimation of herd immunity threshold (*H)*

*H* was calculated as described in [23]:

$$H=1-\left(\frac{1}{R_{0}}\right)$$

and the 95% CI were obtained by the upper and lower estimate of *R*_0_.

All modelling and data analyses were performed in Python.

## Results

### Comparative VZV seroepidemiology in Europe

**Fig 1:**
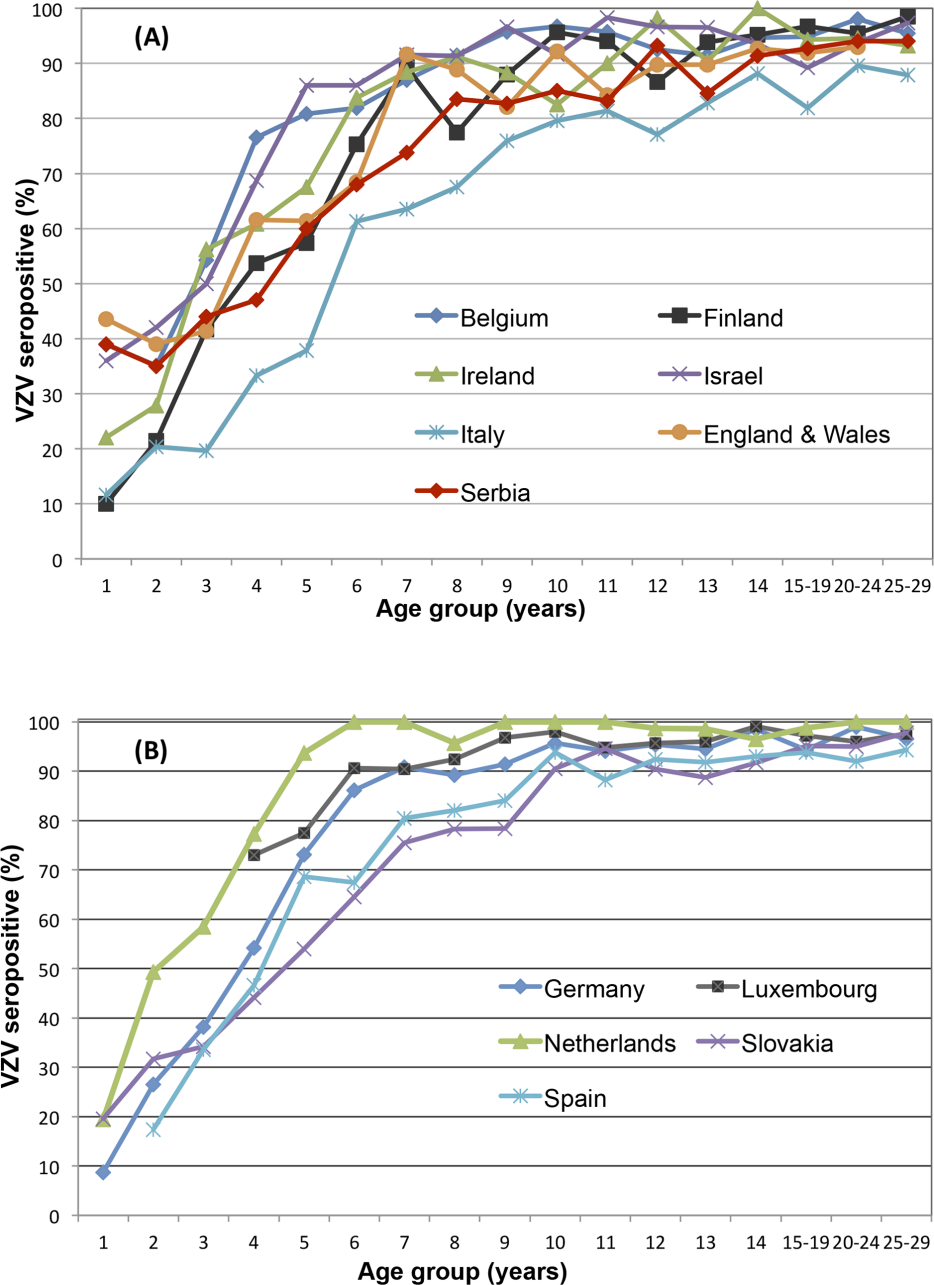
**Age-specific (<30 years) standardized seroprevalence of VZV in Vojvodina, Serbia (2015–16), and in 11 ESEN2 countries where samples had been collected either from residual sera (A), or from population sampling (B), 1995–2003.** The figure is similar but not identical to the original image from [10], and is therefore for illustrative purposes only.

The age-specific VZV seroprofiles of Vojvodina, Serbia and the 11 ESEN2 participant countries are shown in Fig 1.

Antibodies to VZV are acquired in childhood in Europe, but with differing rates of local transmission. Serbia is one of the countries in which anti-VZV are acquired at an earlier age in relation to others (e.g. Italy). Serbia follows the general transmission pattern of most countries, with 41.2% seropositivity in children <5 years (60% at the age of 5 years); nonetheless, the percentages of seropositive 5-9-year-old children and 10-14-year-old adolescents in Serbia (73.6% and 87.5%, respectively) are lower than in other countries apart from Italy. Serbian data for the 5–9 years age group are close to the seroprevalence obtained in Slovakia (69.9%) and Spain (75.9%), while for the 10–14 age group they are similar to the rates in England and Wales (89.7%) (Fig 1 and Table 1).

**Table 1:** Percentage of VZV-seropositive individuals ≤29 years by age group in Vojvodina, Serbia (2015–2016) and in 11 ESEN2 countries (1995–2003)^a^.

Country	Percentage seropositive for VZV by age group (*%*)				
	<5 years	5–9 years	10–14 years	15–19 years	20–29 years
Belgium	51.2	87.4	94.2	94.9	96.8
England & Wales	47.6	78.3	89.7	91.9	92.9^b^
Finland	30.9	77.1	93.1	96.7	97.0
Germany	32.6	86.2	95.6	94.1	97.7
Ireland	41.3	81.7	91.9	94.3	93.8
Israel	49.0	90.6	95.3	89.2	95.1
Italy	21.7	61.1	81.7	81.9	88.8
Luxemburg	73.0^c^	90.1	96.6	97.2	96.8
Netherlands	50.7	97.8	98.8	98.7	100
**Serbia (Vojvodina)**	**41.2**	**73.6**	**87.5**	**92.7**	**94.0**
Slovakia	32.9	69.9	91.2	95.1	96.3
Spain	33.2	75.9	91.7	93.8	93.1

^a^ ESEN2 data have been adopted from [10].

^b^ Samples tested for 20-year-olds only.

^c^ Samples tested for 4-year-olds only.

In Serbia, as in all other countries except Italy, >90% (92.7%) of adolescents aged 15–19 years possess VZV-specific antibodies. Still, a substantial proportion (6%) of young adults aged 20–29 years remains seronegative, matching more closely the susceptibility profiles of Ireland (6.2%), Spain (6.9%), and England and Wales (7.1%). Seronegativity of Serbian females of childbearing age (defined as 15–39 years old) was 5.83% (data not shown).

### Force of VZV infection estimates

#### Estimation of the maximum FOI

**Fig 2:**
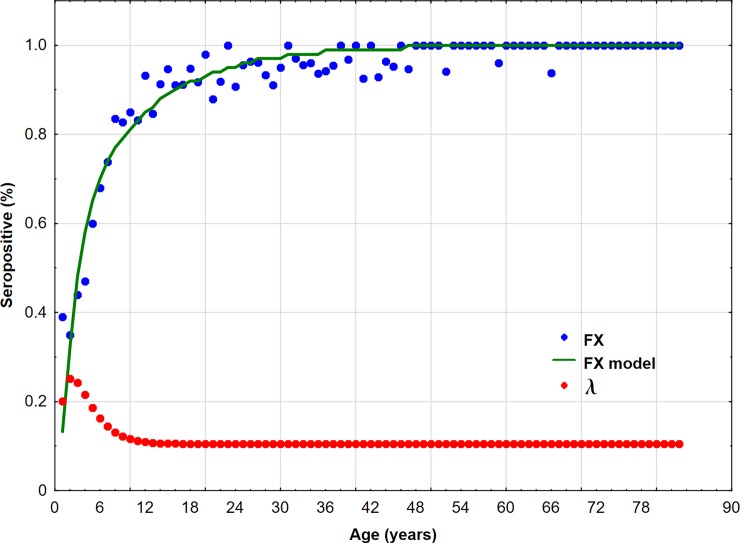
Proportion of seropositive FX, fitted curve FX model and force of infection curve lambda (λ).

The overall fitting of the model was good (*R*^*2*^ = 0.90, residual mean square was 0.16). All parameters *a*, *b* and c were statistically significant, with estimated values as follows: *a =* 0.27, (95% CI: 0.18–0.36), *b* = 0.55, (95% CI: 0.18–0.91), and *c* = 0.11, (95% CI: 0.07–0.14). The maximum FOI *λ*_*max*_ = 0.25 (95% CI: 0.06–0.63) was obtained at $x=\frac{a+bc}{ab}$ = 2.23 years of age (95% CI: 0.57–15.6) (Fig 2).

#### Estimated FOIs, *R*_*0*_ and *H*

In most (9/12) compared countries, the highest FOIs are observed in the 5–9 years age group (Table 2).

**Table 2:** Estimates of age-specific forces of infection (FOI, λ)^a^, basic reproduction number (*R*_*0*_) and herd immunity threshold (*H*) for VZV in Vojvodina, Serbia and in 11 ESEN2 countries^b^.

					Age-specific FOI (*λ*) (95% CI)				
Country	Years of data collection	Age range		Sample size	*λ1* <5 years	*λ2* 5–9 years	*λ3* ≥10 years	*R*_*0*_ (95% CI)	*H* (95% CI)
Belgium	2002	0–71.5		3251	0.31 (0.28–0.34)	0.27 (0.24–0.31)	0.05 (0.03–0.08)	6.47 (5.62–7.55)	84.5 (82.2–86.8)
England & Wales	1996	1–20.9		2032	0.21 (0.19–0.23)	0.23 (0.19–0.26)	0.04 (0.04–0.04)	3.83 (3.32–4.49)	73.9 (69.9–77.7)
Finland	1997–1998	1–79.8		2471	0.16 (0.14–0.18)	0.36 (0.32–0.40)	0.10 (0.07–0.13)	4.85 (3.89–6.04)	79.4 (74.3–83.4)
Germany	1995–1998	0–79		4398	0.19 (0.18–0.20)	0.43 (0.41–0.45)	0.04 (0.03–0.05)	5.46 (5.16–5.76)	81.7 (80.6–82.6)
Ireland	2003	1–60		2430	0.23 (0.21–0.26)	0.29 (0.26–0.33)	0.02 (0.00–0.04)	5.22 (4.53–6.14)	80.8 (77.9–83.7)
Israel	2000–2001	0–79		1543	0.31 (0.28–0.35)	0.28 (0.24–0.34)	0.00 (0.00–0.00)	7.71 (6.01–10.06)	87.0 (83.4–90.1)
Italy	1996–1997	0.1–50		3110	0.10 (0.09–0.11)	0.20 (0.18–0.22)	0.07 (0.06–0.09)	3.31 (2.82–3.83)	69.8 (64.5–73.9)
Luxemburg	2000–2001	4–82		2640	0.33 (0.28–0.37)	0.36 (0.31–0.41)	0.05 (0.020.09)	8.28 (6.74–10.42)	87.9 (85.2–90.4)
Netherlands	1996	0–79		1967	0.35 (0.30–0.40)	0.67 (0.54–0.83)	0.00 (0.00–0.06)	16.91 (11.5–24.18)	94.1 (91.3–95.9)
**Serbia (Vojvodina)**	**2015–2016**	**0.1–83**		**3570**	**0.22 (0.10–0.34)**	**0.20 (0.18–0.22)**	**0.05 (0.03–0.06)**	**4.12 (2.69–7.07)**	**75.7 (62.8–85.8)**
Slovakia	2002	0–70		3515	0.16 (0.14–0.17)	0.25 (0.22–0.29)	0.14 (0.11–0.16)	5.72 (4.72–6.81)	82.5 (78.8–85.3)
Spain	1996	2–39		3590	0.15 (0.14–0.17)	0.33 (0.30–0.35)	0.05 (0.05–0.05)	3.91 (3.53–4.38)	74.4 (71.7–77.2)

^a^ The highest FOI values are highlighted.

^b^ ESEN2 data have been adopted from [10,11].

The exceptions are Belgium, Israel, and Serbia (0.31, 0.31, and 0.22, respectively, in the <5 category). However, the estimated Serbian FOI were only slightly lower (0.20) in the 5–9 years group, where the largest FOI were found in all other countries. Interestingly, the lowest FOI values (0.20) in this age group (5–9 years) were obtained only in Serbia and Italy. The ratio of FOI between the youngest and the oldest age groups is about four in Serbia (0.22/0.05), similarly to several other countries. It is, however, considerably higher in Netherlands (0.35/0.00), Israel (0.31/0.00), or Ireland (0.23/0.02), and lower in Slovakia (0.16/0.14) or Finland (0.16/0.10).

The estimated *R*_0_ and *H* for Serbia, compared to the corresponding data of the 11 ESEN2 participant countries, are also shown in Table 2. The estimated *R*_0_ varies widely within a five-fold difference between the extremes observed in the Netherlands (16.9) and Italy (3.31). Values <5 were estimated in Italy (3.31), England and Wales (3.83), Spain (3.91), Finland (4.85) and Serbia (4.12). However, even if these extremes are ignored, *R*_0_ differs more than twofold, from >8 in Luxemburg to <4 in England & Wales. Serbia is close to the lower end of this range, with an estimated 4.12 new infections originating from a single case. Estimates of *H* are less scattered. Netherlands (94.1) and Italy (69.8) are again positioned at the two extremes. As before, Serbia (75.7%) ranked ninth of 12 countries, next to England and Wales (73.9%), and Spain (74.4%).

## Discussion

The two-tiered mathematical modelling approach revealed that the highest VZV transmission in Vojvodina, Serbia occurs in children of preschool age, with a peak FOI at 2.23 years. A similar pattern was found in Belgium and Israel, whereas in all other ESEN2 countries the largest FOI values were found in the 5–9 years age group. The percentages of seropositive children <5 years were nonetheless higher in Belgium and Israel (~50%) compared to Serbia (41.2%). A comparable value for the peak FOI (λ = 0.282), only at a later age (5 years), was obtained in the Slovenian population a decade ago [13]. The early exposure to VZV and consequent acquisition of natural immunity may be explained by the currently increasing trend of nursery attendance in Serbia, a common practice in countries like Belgium [24].

In most European countries in the pre-vaccine era, the highest age-specific annual incidence rates of varicella, as derived from seroprevalence data, were recently observed in children aged <5 years [3]. The detection of the highest FOIs in the 0.5–4 years age group agrees with the 2015 surveillance data for Vojvodina: the age-specific incidence of varicella was highest in the 1–4 years age group (6482/100 000), a rate 1.4 times higher than in the 5–9 years group (4569/100 000), and three times higher compared to infants ≤1 year of age (2190/100 000)[25].

Kindergarten and preschool mixing patterns constitute the major driving force of virus transmission among 0.5–4 year olds [13,20,26]. National statistics point to an almost 3% increase in the number of children aged 1–6 years that attended childcare or preschool institutions in Serbia in the period 2009–2014; the increase was 12.2% for children <3 years, whilst for children aged 3–5.5 years there was a 0.8% decrease [27]. According to a study conducted by UNICEF in 2012 [28], 48% of children aged ≤5 years attended preschool facilities in Serbia (25% more than in 2005). Increasing nursery attendance might have an impact on varicella disease dynamics through violating the assumption of time homogeneity. To avoid this limitation, longitudinal data or repeated cross-sectional data would be required, but this is not feasible.

A considerable share of susceptible adults has a profound impact on varicella disease burden [29]. Serbian adolescents and young adults were found to be susceptible at somewhat higher percentages (7.3% and 6%, respectively) compared to other ESEN2 countries [4,10]. The proportion (5.83%) of Serbia’s women of childbearing age (15–39 years) is only matched by Ireland (5.4%) and Israel (7.6%) [2,4,10]. Interestingly, the proportion of susceptible women aged 15–49 years (5.46%) in Serbia was almost double to that in Slovenia (2.8%), but almost three times lower than in the neighboring Croatia (16% of women aged 16–45 years) [13,30]. Differences in local assay utilization and study methodology might contribute to these variations. Different assays were used in these studies: Enzygnost (Dade Behring) was employed in Slovenia [13], Virotech in Croatia [30] and EUROIMMUN in Serbia (S1 File). Several papers produced during ESEN2 have shown that results produced by diverse, even well-established assays, or by the same assay in different laboratories, can differ and standardization can help alleviate this problem [9,10]. Furthermore, methodological differences, such as the age limits/definition of reproductive age and time period of sera collection (e.g. 16–45 years between 2007 and 2011 in the Croatia and 15–39 years between 2015 and 2016 in Serbia), could contribute to the differing proportions of susceptible women in these neighboring countries. Collection sites of sera (e.g. urban *vs*. rural areas, from one area only *vs*. geographically representative of the country) could also be contributing factors.

The estimated values of *R*_0_ (4.12) and *H* (75.7%) for Serbia tend to be somewhat lower compared to most ESEN2 countries. The observed international variations of *R*_0_ and *H* may reflect differences in population mixing patterns rather than sampling methodologies; still, they should be taken into account when designing immunization strategies [10]. We employed a WAIFW matrix, considered to mirror the heterogeneous mixing between three age groups, to determine age-specific VZV transmission rates. Using the effective reproduction number *R* that provides a measure of the average number of secondary cases that may occur in a partially immune population in addition to *R*_0_, could have enhanced our analysis [22]. Santermans *et al*. [11] recently used *R* to determine factors affecting the between-country heterogeneity in *R*_0_ among ESEN2 participants. Positive associations with *R*_0_ pertained to childhood immunization coverage, kindergarten attendance, population density and average living area per person, while income inequality and poverty, breast feeding, and the proportion of children <14 years showed negative associations. Xiao *et al*. [31] demonstrated that physical contacts among school age children are most relevant to VZV transmission in the five European countries involved in the POLYMOD survey.

Recommendations of VZV serologic screening of women of childbearing age without a history of varicella and immunization of seronegatives, prior to pregnancy or post-partum, are widely accepted policies [32]. The recent Serbian Rulebooks [7,33] recommend serologic testing for VZV in pregnancy and passive immunisation of exposed seronegative pregnant women. Nevertheless, anti-VZV testing and immunization of seronegative non-pregnant women of childbearing age are not officially recommended. Current Serbian immunization policy [6,7] prioritises the immunization of high-risk groups although past experience in most EU/EEA countries shows that this strategy was accompanied by lower vaccine coverage rates [13] and had no potential to interrupt VZV transmission compared to universal childhood immunization [4,24]. Universal childhood varicella immunization proved to be cost-effective and efficient in reducing disease burden [1,2,24]. If such a policy were adopted in Serbia, an accelerated two-dose vaccine schedule, *vs*. one dose could enhance vaccine coverage and diminish the risk of “breakthrough” varicella [24]. The standard schedule (first dose at 12–24 months and second dose at 3–7 years) would allow immunization with combined measles-mumps-rubella-varicella (MMRV) vaccine since the MMR vaccine is already obligatorily scheduled at seven years of age [6,7]. In either case, the first dose of the vaccine should be administered ideally at 12–18 months, given that the maximal FOI was observed at 2.23 years. Preferably administration of both doses of varicella vaccine should be scheduled during the second year of life in order to increase vaccine coverage, reduce the number of VZV susceptible children and minimize the risk of breakthrough disease due to the relatively high rate of primary vaccine failure [34]. In this case, there would be no need for a further visit to the doctor in view of the two planned revaccinations in the second year of life (combined vaccine against diphtheria, tetanus, pertussis, haemophilus influenzae B and polio as well as pneumococcal conjugate vaccine) and given that varicella vaccine can be given simultaneously [7]. Considering the substantial share of susceptible adolescents, targeted catch-up campaigns would be required [10,24].

Despite predictions, most studies in countries with successful coverage rates after the implementation of universal varicella childhood immunization have not shown an accompanying increase in HZ incidence [12,24]. On the contrary, HZ incidence was found to be reduced; the hypothesis that gained ground was that the percentage of individuals harboring latent VZV declined due to the decreased incidence of varicella [24]. *Baxter at al*. [35] established a ∼40% decrease of HZ incidence in vaccinated children over the first 14 years after varicella vaccination compared with unvaccinated children of the same age who acquired varicella naturally. This evidence further reinforces the introduction of universal varicella immunization in childhood.

Taking into account the estimated value of *H* (76%) and varicella vaccine effectiveness, the recommended vaccine coverage should be at least as recommended by WHO (≥ 80%); a lower coverage may shift varicella cases to older age with consequent increases in complications and mortality [29]. However, recent, below the 95% target MMR vaccination coverage in Serbia [36], underscores the need for creating stable preconditions for the introduction of MMRV vaccine. Re-introduction of both varicella and HZ surveillance to follow up on the impact of the current risk-group and future routine childhood varicella immunization strategies on VZV disease burden is also necessary.

## Conclusions

Considering that varicella stems from a common airborne infection, it is not surprising that Serbian seroprevalence data from Vojvodina, along with estimated key epidemiological parameters, generally fit into the dynamic VZV transmission patterns of most European countries in the pre-vaccine era. However, the Serbian VZV seroprofile is characterized by most intense viral transmission in pre-school children aged <4 years and epidemiologically relevant gaps in immunity of young adults. Obtained over a decade ago, the results of the ESEN2 study continue to serve as a cornerstone for comparisons of VZV epidemiological parameters in Europe. The ability of each country to meet the necessary financial preconditions and provide optimal immunization coverage, varies considerably. The data collected in this survey provide an important basis for analysing costs and benefits, priorities with respect to other vaccine-preventable illnesses in light of financial constraints and vaccine acceptability. Further studies for the quantification of the burden of varicella and HZ in Serbia are deemed necessary to design the most appropriate immunization policy.

## Supporting information

S1 File The Serbian 2015–2016 VZV serosurvey. (DOCX)
