# Supplementary material for: Varicella zoster virus transmission dynamics in Vojvodina, Serbia
Source: PLoS One. 2018 Mar 5;13(3):e0193838. doi: 10.1371/journal.pone.0193838 (PMC5837184; doi:10.1371/journal.pone.0193838)
Supplement: S1 File — (DOCX) [file pone.0193838.s001.docx]

**S1 File. The Serbian 2015-2016 VZV serosurvey.**

A multicenter, cross-sectional serosurvey was carried out between April 2015 and March 2016 to assess the varicella zoster virus (VZV) seroprevalence in the Autonomous Province of Vojvodina, Serbia. Vojvodina is a northern Serbian province in central Europe that covers an area of 21,506 km^2^. The province has almost two million inhabitants (1,931,809), comprising 26.9% of the population of Serbia, excluding Kosovo [1]. The share of elderly in the population is substantial (40.2% of the population is >50 years), while the proportion of children is low (14.5% of the population is comprised of children aged <15 years). Health institutions provide medical services predominantly to urban and suburban populations, but the share of citizens living in rural areas (40.6%) is significant [2].

Overall 3570 serum samples (males: 50%) were collected. The age of respondents ranged between 29 days and 83 years. Overall 52 sera samples belonged to children <6 months of age. Samples from females of reproductive age (15-39 years old) corresponded to 38.5% of samples collected from women. The serosurvey was based on the ESEN2 methodology to allow for international comparisons [3]. Residual sera had been collected from patients referred for routine laboratory testing (for check-ups and diagnostic purposes) in health care centers and hospitals in each of the seven districts of Vojvodina. Sera were thus geographically representative of the province. In total, 1449 and 2121 sera were collected from residents of rural and urban areas, respectively. The stratification of the serum bank at the municipality level was according to the latest census [1]. The study protocol required written informed consent of participants (or their parents/legal guardians for children <15 years old). A blood sample was drawn after discussing the health history of each respondent. Immunocompromised individuals and recent recipients of blood and blood products were excluded. Samples were anonymous as data for each participant included only the following: sex, age, area of residence and sample collection date. The study was approved by the Medical Ethics Committee of the Institute of Public Health (IPH) of Vojvodina, in accordance to the Declaration of Helsinki of 1975, as revised in 2008.

Sera were stored at -20°C until testing with anti-VZV ELISA (IgG) [EUROIMMUN AG, Germany] according to the manufacturer’s guidelines, at the Virology laboratory of IPH of Vojvodina. Obtained results were standardized into ESEN2 units according to the ESEN2 methodology [3]. The ESEN2 VZV standardization panel (148 samples) provided by the reference laboratory (Instituto de Salud Carlos III, Madrid, Spain) was tested at the beginning and mid-way through the testing of the serum bank [4]. The derived standardization equation was used to convert the local results of the serosurvey into standardized units by applying the reference laboratory cut-offs [5]. Results were subsequently reclassified qualitatively as negative, positive or equivocal in standardized ESEN2 units, with equivocals (low positives) included as seropositives.

**References**

Statistical Office of the Republic of Serbia. 2011 Census of Population, Households and Dwellings in the Republic of Serbia. [In Serbian/English]. <http://pod2.stat.gov.rs/ObjavljenePublikacije/Popis2011/Nacionalna%20pripadnost-Ethnicity.pdf>. Accessed 20 May 2017.

Statistical Office of the Republic of Serbia. Statistical year book of Republic of Serbia, 2015. [In Serbian/English]. Available from: <http://webrzs.stat.gov.rs/WebSite/userFiles/file/Aktuelnosti/StatGod2015.pdf>. Accessed 18 December 2016.

1. Nardone A, de Ory F, Carton M, Cohen D, van Damme P, Davidkin I, et al. The comparative sero-epidemiology of varicella zoster virus in eleven countries in the European region. Vaccine. 2007;25(45):7866-7872. doi:10.1016/j.vaccine.2007.07.036
2. de Ory F, Echevarría JM, Kafatos G, Anastassopoulou C, Andrews N, Backhouse J, et al. European Seroepidemiology Network 2: standardisation of assays for seroepidemiology of varicella zoster virus. J Clin Virol. 2006;36(2):111-118.

Kafatos G, Andrews N, Nardone A. ESEN2 project. Model selection methodology for inter-laboratory standardisation of antibody titres. Vaccine. 2005;23(42):5022–5027.
